# Differential Transcript Profiles of MHC Class Ib(Qa-1, Qa-2, and Qa-10) and *Aire* Genes during the Ontogeny of Thymus and Other Tissues

**DOI:** 10.1155/2014/159247

**Published:** 2014-04-16

**Authors:** Breno Luiz Melo-Lima, Adriane Feijó Evangelista, Danielle Aparecida Rosa de Magalhães, Geraldo Aleixo Passos, Philippe Moreau, Eduardo Antonio Donadi

**Affiliations:** ^1^Basic and Applied Immunology Program, Division of Clinical Immunology, Department of Medicine, Faculty of Medicine of Ribeirão Preto, University of São Paulo, Avenida Bandeirantes 3900, 14049-900 Ribeirão Preto, SP, Brazil; ^2^Commissariat à l'Energie Atomique et aux Energies Alternatives, Institut des Maladies Emergentes et des Therapies Innovantes, Service de Recherches en Hémato-Immunologie, Hôpital Saint-Louis, 1 avenue Claude Vellefaux, Bâtiment Lailler, 75475 Paris Cedex 10, France; ^3^Université Paris-Diderot, Sorbonne Paris-Cité, UMR E5, Institut Universitaire d'Hématologie, Hôpital Saint-Louis, 1 avenue Claude Vellefaux, 75475 Paris Cedex 10, France; ^4^Molecular Immunogenetics Group, Department of Genetics, Faculty of Medicine of Ribeirão Preto, University of São Paulo, Avenida Bandeirantes 3900, 14049-900 Ribeirão Preto, SP, Brazil

## Abstract

Qa-2 and Qa-1 are murine nonclassical MHC class I molecules involved in the modulation of immune responses by interacting with T CD8^+^ and NK cell inhibitory receptors. During thymic education, the *Aire* gene imposes the expression of thousands of tissue-related antigens in the thymic medulla, permitting the negative selection events. Aiming to characterize the transcriptional profiles of nonclassical MHC class I genes in spatial-temporal association with the *Aire* expression, we evaluated the gene expression of *H2-Q7*(Qa-2), *H2-T23*(Qa-1), *H2-Q10*(Qa-10), and *Aire* during fetal and postnatal development of thymus and other tissues. In the thymus, *H2-Q7*(Qa-2) transcripts were detected at high levels throughout development and were positively correlated with *Aire* expression during fetal ages. *H2-Q7*(Qa-2) and *H2-T23*(Qa-1) showed distinct expression patterns with gradual increasing levels according to age in most tissues analyzed. *H2-Q10*(Qa-10) was preferentially expressed by the liver. The *Aire* transcriptional profile showed increased levels during the fetal period and was detectable in postnatal ages in the thymus. Overall, nonclassical MHC class I genes started to be expressed early during the ontogeny. Their levels varied according to age, tissue, and mouse strain analyzed. This differential expression may contribute to the distinct patterns of mouse susceptibility/resistance to infectious and noninfectious disorders.

## 1. Introduction

Immune tolerance has been assigned to two broad categories according to the places where it occurs, that is, central and peripheral tolerance [1]. It is well known that nonclassical Major Histocompatibility Complex class I molecules (MHC Ib), like HLA-G and HLA-E, are associated with the regulation of immune responses in the periphery; however, little is known regarding the effect of these molecules at the central level. Human MHC Ib molecules exhibit restricted tissue distribution and do not have an important role in antigen presentation, and the coding regions of the respective genes have relatively low variability, particularly at exons that code major functional regions of the molecules [2].

In mice, two molecules have been described to be functional homologues of HLA-G and HLA-E: Qa-2 and Qa-1, respectively. Like humans, Qa-2 and Qa-1 molecules are involved in the regulation of immune responses and are encoded by the* H2*-*Q7/H2-Q9* (also called* Ped gene—preimplantation embryo development gene*) and* H2-T23* genes, respectively, both located in Histocompatibility Complex-2 (H-2) [3, 4].

The H-2 spans approximately 4 Mb of chromosome 17 (23.0 cM, cytoband B-C) and contains 3 major classes of highly polymorphic gene sets: class I (*H-2*-K,* H-2*-D, Q, and* H-2*-T18 genes), class II (*H-2*-I genes), and class III (*H-2*-S genes). These genes are involved in many immunological processes, including graft rejection, immune response, antigen presentation, and complement component [5]. The number of class I genes, their organization, structural characteristics, and their patterns and levels of expression differ from species to species [3]. In general, C57BL/6 and BALB/c mice are considered in two different haplotypes:* H-2* ^b^ and* H-2* ^d^, respectively [4] (http://www.informatics.jax.org/).

The murine Qa-2 genes map to the H-2Q region, between the classical class I H-2D locus and the H-2TL cluster of class lb sequences. This locus encodes a variable number of class Ib genes in different strains [3]. Strains expressing high (Qa-2^high^), medium (Qa-2^med^), low (Qa-2^low^), and no Qa-2 (Qa-2^nul^) were identified [3]. In general, BALB/c strain is considered to be Qa-2^med^ having two Qa-2 genes (Q6^d^ and Q7^d^). In C57BL/10, considered to be Qa-2^high^, four genes with the Qa-2-coding properties are located within the Q regions: Q6^b^, Q7^b^, and, additionally, Q8^b^ and Q9^b^ [3].

Qa-2 is a 40-kDa glycoprotein found as membrane-bound and soluble isoforms generated by alternative splicing [3, 6, 7]. Qa-2 is expressed mostly by lymphoid-derived cells and plays an important role in controlling growth and murine fetal development and, like HLA-G, the molecule is related to the protection of the fetus by inhibiting maternal NK cell-mediated lysis [8–10].

Qa-1 is a 48 kDa cell surface glycoprotein currently found in association with**β**2-microglobulin [4, 11]. Its surface expression is found in lower levels virtually in all tissues and is increased in activated hematopoietic, T, B, and antigens presenting cells [4]. Qa-1 is involved in suppression of CD4^+^ T cell and NK cell responses through a preferential interaction with inhibitory CD94/NKG2A receptors [4, 12]. The suppression and modulation of autoreactive T CD4^+^ and B clones is mediated by T CD8^+^ regulatory cells that recognize autoantigens presented by Qa-1 molecules [4, 12, 13]. These regulatory cells perform perforin-mediated lysis and production of immunomodulatory cytokines such as TGF-**β** and IL-10 [4, 12, 13].

Another murine MHC class Ib molecule involved in the modulation of the immune system is Qa-10, encoded by the* H2-Q10* gene. Qa-10 is synthesized at high levels by the liver parenchymal cells and is easily detectable in the serum as a high multivalent complex [14]. It has been suggested that Qa-10 liver cell expression may be responsible for the relative lack of immunogenicity of liver transplants and better acceptance of liver allografts [15, 16].

It has been postulated that the regulatory role played by MHC class Ib molecules is an additional mechanism that controls autoimmune reactions in peripheral autoreactive lymphocytes that escaped from central tolerance during the ontogeny of the thymus [13]. Indeed, the mechanisms of central tolerance that occur during thymus development are critical processes for the prevention of autoimmunity during the fetal and neonatal periods [17, 18]. This process characterizes the negative selection that purges the T cell repertoire of self-reactive clones through clonal deletion, inactivation, or deviation [18, 19]. The medullary thymic epithelial cells (mTECs) are primarily associated with negative selection through the expression of a wide array of tissue-restricted antigens (TRAs), a process also termed promiscuous gene expression (PGE) [20–22].

PGE is greatly dependent on the* Aire* gene* (autoimmune regulator)* [18, 21]. A mutation in this gene leads to a severe multiorgan autoimmune polyglandular syndrome type I (APS 1, also called APECED), in both mice and humans [17, 18]. It has currently been reported that most genes encoding promiscuously expressed TRAs in the thymus are regulated by a single* Aire* gene product, which is involved in a multiprotein complex transcriptional process responsible for transcription initiation, modifications of chromatin, transcriptional regulation of mRNA during the productive elongation phase, and regulation of alternative splicing events of the pre-mRNA [23, 24].

Interestingly, the expression of several MHC class Ib molecules has been reported on certain thymic cell subpopulations in mice and humans. Qa-2 expression has been used to identify functionally competent medullary thymocytes [25–27]. HLA-G is highly expressed on mTECs and stromal cells at the corticomedullary junction, and high levels of soluble HLA-G are observed in the thymus medullary compartment [28]. Qa-1 is expressed on the surface of hematopoietic cells responsible for the positive selection of Qa1-restricted CD8^+^ T cells, allowing the maturation and selection of potentially self-reactive T CD8^+^ regulatory cells [4, 11]. In addition to expressing immunoregulatory MHC Ib molecules, the thymus is the primary site of* Aire* expression, which is characteristically synthesized by mTEC subsets, intrathymic dendritic cells, and thymic macrophages [18].

During thymus ontogeny in the fetal stage, the expression of MHC class I and class II molecules is crucial for the education and selection of the repertoire of lymphocytes [29]. The early fetal thymus from E13.5 to E17.5 day* p.c.* is primarily composed of a homogeneous population of double-negative (DN) CD4^−^/CD8^−^ precursor T cells. The gradual acquisition of the CD4 antigen occurs around E18.5 day, with the positive selection process starting thereafter [30]. This period (E16.5–E18.5) coincides with the onset of V(D)J recombination of T cell receptor in DN thymocytes and with the beginning of promiscuous gene expression, in which the* Aire* gene has a well-recognized role [31]. From the E18.5 day p.c., thymocytes gradually gain the phenotypic markers resembling the T CD4^low^ lymphocyte precursors in adults. These events allow the recognition and interaction of the T cell receptor with the MHC-peptide complex, allowing the occurrence of positive and negative selection in these cells [30]. The negative selection process occurs during perinatal ages and extends during the first 15 days after birth [17, 32]. This period is a critical stage in thymus ontogeny that is related to the mechanisms operating in the prevention of autoimmune processes [17, 32].

Considering that (i) many immunomodulatory MHC class Ib molecules are expressed in the thymus, (ii) Aire is a paradigmatic thymic immunomodulatory molecule that may have the ability to induce the expression of other molecules involved in the regulation of immune responses; (iii) many immunomodulatory molecules have differential temporal expression during thymus ontogeny, and (iv) different experimental strains may show distinct profiles of immunoregulatory molecules, we hypothesized that nonclassical MHC class I gene expression, such as* H2-Q7*(Qa-2),* H2-T23*(Qa-1), and* H2-Q10*(Qa-10), could be temporally related to* Aire* expression, participating in the occurrence of the central tolerance process and extending through the postnatal thymus development. To achieve this goal, we assessed the pattern of* Aire* expression and also the expression of nonclassical MHC class I genes by evaluating the temporal transcript profiles of* Qa1, Qa2, Qa10*, and* Aire* in the thymus of C57BL/6 and BALB/c mice, starting from the embrionary (E14.5 days) period and continuing till adulthood (60 days). The temporal transcript profile of these genes was also evaluated in some lymphoid and nonlymphoid tissues (spleen, liver, and gut) and in immunologically privileged sites (brain and placenta) to correlate with thymus findings.

## 2. Material and Methods

### 2.1. Animals

C57BL/6 and BALB/c mice were bred in an isolated cage provided with 0.45 *μ*m pore size air filter. To obtain an accurate day of gestation, the presence of a post coitum vaginal plug observed at 7:00 am was considered to be day zero. Fetuses were surgically collected from the uterus, and p.c. age was confirmed according to the morphological characteristics of each developmental phase [32]. Tissue samples were obtained in triplicate from (i) fetuses aged E14.5, E15.5, E16.5, E17.5, E18.5, E19.5, and E20.5 days of gestation, (ii) newborns aged 1, 5, 10, and 15 days, and (iii) adults aged 45 and 60 days. For each age were used at least 3 different animals. For tissue harvesting all tissues were washed in saline solution and then processed. For thymus both lobes were processed. For spleen, liver, gut, placenta, and brain only the same portions of tissues were analyzed. There was no separation between hematopoietic cells and parenchyma. Experimental procedures followed ethical guidelines under strict guidance and approval from the University of São Paulo Ethics Committee for Animal Experimental Research (Protocol number 043/2009).

### 2.2. RNA Extraction

After tissue isolation, total RNA samples were obtained by maceration of each tissue in TRizol reagent using a Potter homogenizer according to the manufacturer's instructions (Invitrogen, Carlsbad, CA) and treated with DNAse (deoxyribonuclease I amplification grade, Invitrogen). RNA integrity was checked by the presence of the 28S and 18S bands in 1.5% agarose gel, and only protein-free, phenol-free, and undegraded RNA species were used, as determined by UV spectrophotometry.

### 2.3. Analysis of Expression by Real-Time PCR

Total RNA isolated from samples was reverse-transcribed to cDNA using the High Capacity cDNA Transcription Kit (Applied Biosystems, Foster City, CA), following the manufacturer's instructions. cDNA amplification was initially carried out in a total volume of 25 *μ*L, corresponding to 500 ng of the initial RNA.

We assessed the expression of the* H2-Q7*(Qa-2),* H2-T23*(Qa-1),* H2-Q10*(Qa-10), and* Aire* genes by quantitative real-time PCR using TaqMan Probe-Based Gene Expression Analysis (Applied Biosystems) in a total volume of 10 *μ*L containing 75 ng total RNA, 5 *μ*L TaqMan PCR Universal Master Mix (Applied Biosystems), and 0.5 *μ*L TaqMan Gene Expression Assays. An ABI System Sequence Detector 7500 (Applied Biosystems) was used with the following regimen of thermal cycling: stage 1—1 cycle for 2 minutes at 50°C; stage 2—1 cycle for 10 minutes at 95°C; stage 3—40 cycles for 15 seconds at 95°C, followed by the last cycle for 1 minute at 60° and 25 seconds at 72°C. Gene expression was normalized relatively to the TaqMan endogenous controls (Applied Biosystems), using* glyceraldehyde-3-phosphate dehydrogenase* and**β*-actin* genes. The relative quantification of transcript levels at the different ages and tissues was performed by the comparative 2^−ΔΔCt^ method for each different analysis using the ΔCt minimum as control sample. Each sample was tested in triplicate. The TaqMan Inventoried Assays and TaqMan Gene Expression Assay reference are listed as follows:* H2-Q7*: Mm00843895_m1;* H2-Q10*: Mm01275264_m1;* H2-T23*: Mm00439246_m1;* Aire*: Mm00477461_m1;* GAPDH*: 4352339E;* ACTB*: 4352341E.

Statistical analysis was performed using one-way ANOVA followed by the Bonferroni multiple comparison test for analysis of gene expression profiles in different tissue samples. To compare age-related tissue samples between lineages, we used the Student's *t*-test. The analyses involving several variables (type of tissue, age, and genes) were performed using the two-way ANOVA Bonferroni's multiple comparison test with the aid of the Graphpad Prism V.5 software (San Diego, CA, http://www.graphpad.com/prism/). The Pearson product-moment correlation coefficient was calculated using R software version 2.14.0 (http://www.r-project.org/). *P* < 0.05, *P* < 0.01, and *P* < 0.001 were considered statistically significant.

## 3. Results

### 3.1. Analysis of MHC Class Ib and* Aire* Transcripts in Different Tissues of C57BL/6 Mice

**Figure 1:**
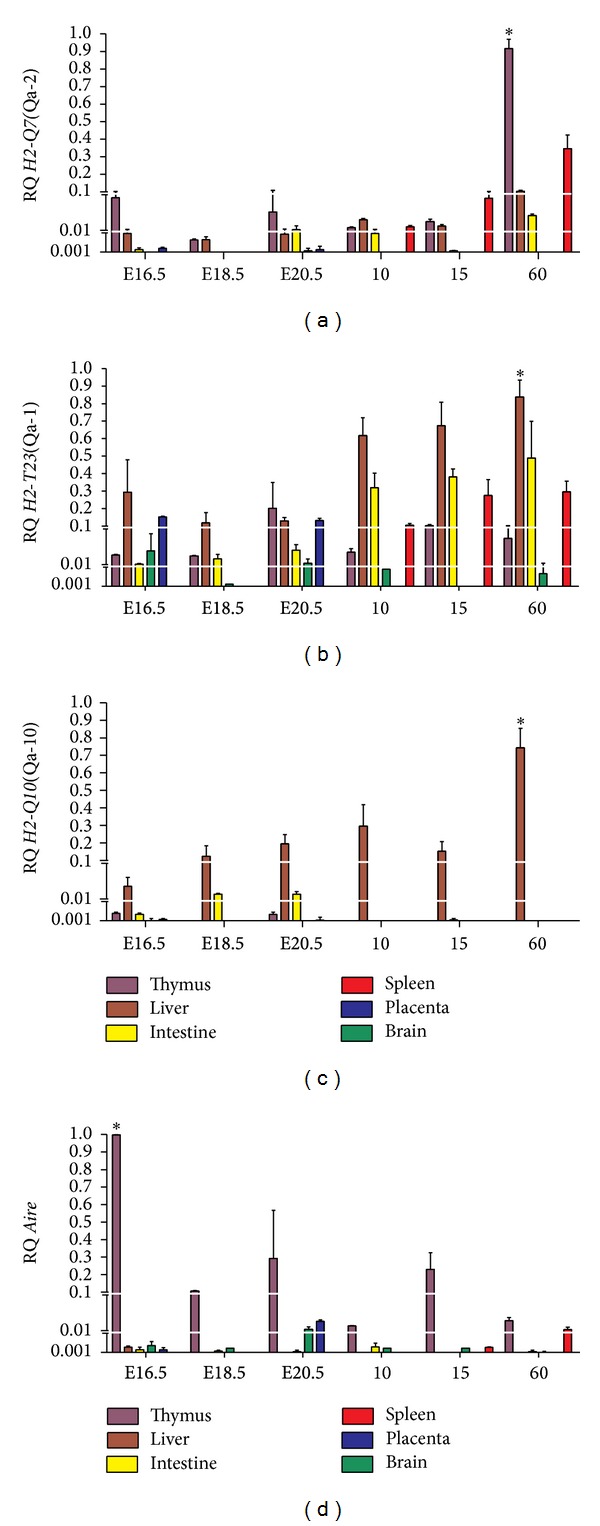
Analysis of MHC class Ib and* Aire* transcripts in different tissues of C57BL/6 mice. Relative quantification (RQ) of messenger RNA for (a)* H2-Q7*(Qa-2), (b)* H2-T23*(Qa-1), (c)* H2-Q10*(Qa-10), and (d)* Aire* is representative of the mean values of 2^−ΔΔCt^. Tissue samples were obtained in triplicate from different animals for each age analyzed. Each experiment was independently performed at least three times. Data were analyzed statistically by two-way ANOVA followed by the Bonferroni multiple comparison test. Values close to the level of significance are marked with (∗).

Aiming to discriminate the primary organ of gene expression, we first compared each individual's gene transcript levels among the different tissues analyzed. We found that the* H2-Q7* transcripts were primarily observed in thymus, followed by spleen and liver at all ages, being higher in adult thymus compared with other tissues along at different ages (*P* < 0.01) (Figure 1(a)). The transcript levels of* H2-T23* were homogeneously expressed among all tissues, showing higher levels only in liver at adult ages (*P* < 0.01) (Figure 1(b)).* H2-Q10* proved to be liver-specific, gradually increasing with age (*P* < 0.001).* H2-Q10* expression was also detected in the fetal thymus and intestine (Figure 1(c)). Abundant levels of* Aire* transcripts were observed in the thymus during fetal ages, with a peak occurring at day E16.5 (*P* < 0.001), although, at reduced levels,* Aire* transcripts were observed at all postnatal ages. In addition, restricted levels of this gene were observed in brain and placenta (during fetal periods) and in spleen of adult mice (Figure 1(d)). In this analysis the gene transcript levels of each gene singly were compared between all tissues and ages.

### 3.2. Comparisons of Gene Expression Profiles of MHC Class Ib and* Aire* during Development of Thymus, Lymphoid, and Nonlymphoid Tissues in C57BL/6 Mice

**Figure 2:**
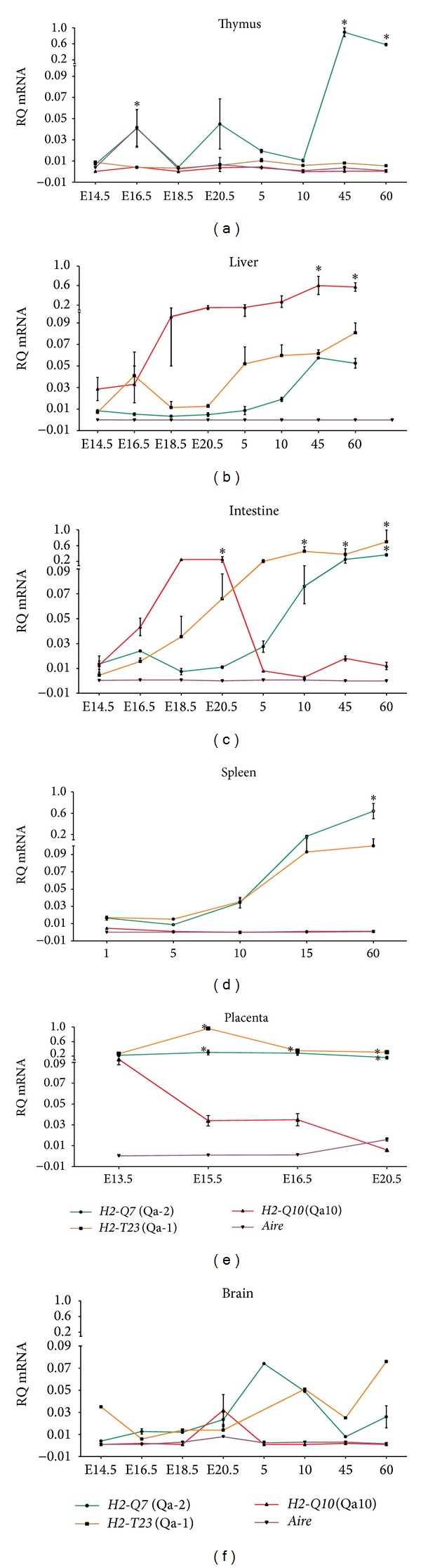
Comparisons of gene expression profiles of MHC class Ib and* Aire* during development of thymus, lymphoid, and nonlymphoid tissues in C57BL/6 mice. Relative quantification (RQ) obtained from (a) thymus, (b) liver, (c) intestine, (d) spleen, (e) placenta, and (f) brain is representative of the mean values of 2^−ΔΔCt^. obtained from comparisons of the four genes in each tissue independently. Tissue samples were obtained in triplicate from different animals for each age analyzed. Each experiment was independently performed at least three times. Data were analyzed statistically by two-way ANOVA followed by the Bonferroni multiple comparison test. Values close to the level of significance are marked with (∗).

To compare the transcription pattern during organ ontogeny, we analyzed each transcript in isolated tissues according to age. During fetal thymus development, both* H2-Q7* and* Aire* transcripts were significantly increased at E16.5 days in comparison to* H2-Q10* and* H2-T23* at all ages (*P* < 0.001). In addition,* H2-Q7* was the most widely expressed gene throughout the period of thymus development, reaching significance at E16.5, 45, and 60 days (*P* < 0.001). In adult thymus,* Aire* transcripts were observed at levels similar to* H2-Q10* and* H2-T23* (Figure 2(a)). In liver samples,* H2-Q10* expression was high and increased with aging (Figure 2(b)); however, in gut samples, these transcripts were elevated during fetal and perinatal ages (*P* < 0.05). During postnatal periods, the* H2-T23* gene was the most expressed (*P* < 0.001), followed by* H2-Q7* (*P* < 0.01) (Figure 2(c)). In the spleen, a gradual transcript increase was observed for* H2-Q7* and* H2-T23*.* H2-Q7* was significantly higher in spleen samples from 60-day-old animals (*P* < 0.001). A diminished expression of* Aire* was observed in spleen samples at postnatal ages (Figure 2(d)). Interestingly, during the evolution of pregnancy, the* H2-T23* transcripts levels were higher than* H2-Q7* at day E15.5 (*P* < 0.001) and day E16.5 (*P* < 0.001), and at day E20.5 (*P* < 0.05) in placenta samples (Figure 2(e)). Overall, MHC class Ib transcripts were observed at very restricted levels in brain at all ages analyzed, except for a faint expression of* H2-Q7* transcripts during the perinatal period (Figure 2(f)).

### 3.3. Individual Transcript Patterns of* H2-Q7*(Qa-2),* H2-T23*(Qa-1),* H2-Q10*(Qa-10), and* Aire* throughout Development in C57BL/6 Mice

The individual values of the transcripts for* H2-Q7*,* H2-Q10*,* H2-T23,* and* Aire* throughout the development are shown in Table 1 and are representative of the mean values of 2^−ΔΔCt^.

In the thymus, transcript levels of* H2-Q7* gradually increased with age, reaching significance in mice aged 1, 45, and 60 days compared to other ages.* H2-T23* expression was increased in animals aged 1, 5, 15, and 45 days compared to fetal and adult ages. Although it is considered to be liver-specific,* H2-Q10* expression was surprisingly increased during the fetal period of thymus development at days E15.5, E16.5, E19.5, and E20.5 and during the perinatal period at days 1 and 5.* Aire* expression was significantly increased during fetal thymus development, primarily at E16.5 and E17.5.* Aire* expression was maintained at reduced levels during postnatal periods in the thymus of newborn and adult animals.

In the liver, gene expression profiles of* H2-Q7*,* H2-T23*, and* H2-Q10* were detected at low levels at fetal and perinatal ages, increasing after day 5. Postnatal expression of* H2-Q7* and* H2-Q10* peaked at days 45 and 60, whereas* H2-T23* expression continued to be elevated from day 5 to 60.* Aire* gene transcription was not detected during liver development.

In the gut, the expression of* H2-Q7* and* H2-T23* was increased in the postnatal period, primarily from day 15 to day 60. In contrast,* H2-Q10* transcripts peaked at late fetal periods on days E19.5 and E20.5.* Aire* expression was observed at all ages, exhibiting significant peak expression at day E14.5 and at perinatal day 5.

Due to the late formation of the spleen, the tissue samples were obtained only during postnatal periods.* H2-Q7* and* Aire* showed a closely similar expression pattern, exhibiting peak levels at day 60. In contrast,* H2-Q10* transcripts peaked at day 1. No significant differences in* H2-T23* expression were observed.

In placenta samples, the gene expression profiles of* H2-Q7* and* H2-T2*3 showed no significant differences from day E14.5 to day E20.5. On the other hand, the expression of* H2-Q10* peaked at day E14.5 and the expression of* Aire* peaked at day E20.5.

In the brain, analysis of the expression of* H2-Q7, H2-T2*3,* H2-Q10*, and* Aire* in fetal and adult mice showed no significant differences.

### 3.4. Comparison of Gene Expression Profiles between C57BL/6 and BALB/c Mice

**Figure 3:**
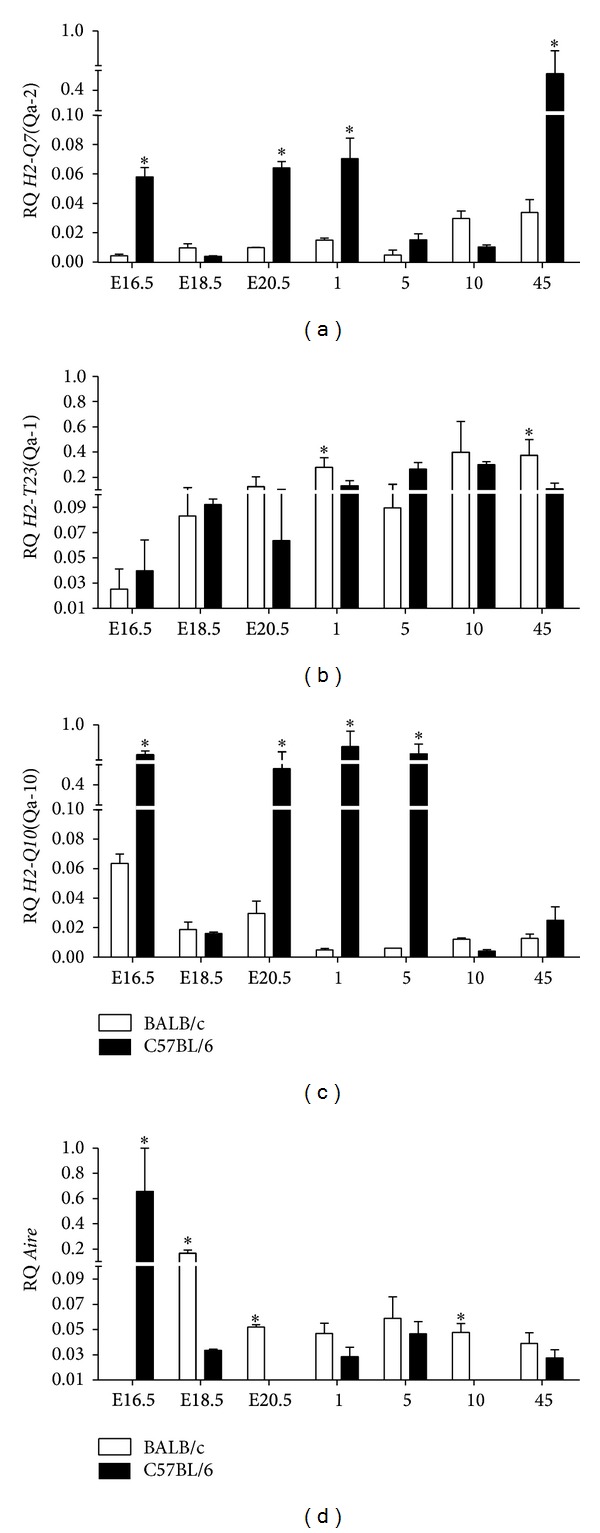
Comparison of gene expression profiles during thymus development between C57BL/6 and BALB/c mice. Relative quantification (RQ) of messenger RNA for (a)* H2-Q7*(Qa-2), (b)* H2-T23*(Qa-1), (c)* H2-Q10*(Qa-10), and (d)* Aire* is representative of the mean values of 2^−ΔΔCt^. Tissue samples were obtained in triplicate from different animals and lineages for each age analyzed. Each experiment was independently performed at least three times. Data were analyzed statistically by the Student's *t*-test. Values close to the level of significance are marked with (∗).

Compared with BALB/c mice, the C57BL/6 mouse thymus showed (i) higher transcript levels for* H2-Q7* and* H2-Q10* during fetal and postnatal development; (ii) increased levels of* H2-Q7* on the following days: E16.5 (*P* = 0.0142), E20.5 (*P* = 0.0063), 1 (*P* = 0.0037), and 45 (*P* < 0.0001) (Figure 3(a)); (iii) closely similar* H2-T23* transcript levels, except at days 1 and 45, when* H2-T23* expression levels were significantly increased in BALB/c mice (*P* = 0.01 and *P* = 0.0058, resp.) (Figure 3(b)); (iv) increased levels of* H2-Q10* mRNA in mice aged E16.5 (*P* < 0.01), E20.5 (*P* < 0.01), 1 (*P* < 0.001), and 5 postnatal days (*P* < 0.001) (Figure 3(c)); (v) a peak of* Aire* transcripts at day E16.5 (*P* < 0.001), while in BALB/c mice this peak occurred at day E18.5 of development (*P* = 0.0001). Overall, BALB/c mice expressed higher levels of* Aire* in the thymus at all other ages evaluated in this study, reaching significance at fetal E18.5 (*P* = 0.0001) and E20.5 days (*P* = 0.0318) and at 10 days after birth (*P* = 0.0004) (Figure 3(d)).

Regarding other organs (liver, gut, spleen, placenta, and brain), C57BL/6 mice expressed considerably higher levels of* H2-Q7* than BALB/c mice. In addition,* H2-Q7* transcripts in fetal E17.5 liver (*P* = 0.0125), in E16.5 and E18.5 placentas (*P* = 0.00137), in 10 day spleen (*P* = 0.0146), and in 15-day gut (*P* = 0.0002) were higher in C57BL/6 mice (data not shown).* H2-T23* transcripts were more abundant in BALB/c mice at most ages and in most organs compared to C57BL/6 mice, being significantly increased at E17.5, E18.5, and E20.5 day in the fetal liver (*P* = 0.0057, *P* = 0.0196, and *P* = 0.0174, resp.) and at day 10 in the gut (*P* < 0.001). No significant differences in* H2-T23* levels were observed in the spleen of these mice (data not shown).* H2-Q10* was significantly increased in fetal liver and fetal gut of C57BL/6 mice aged E20.5 (*P* = 0.0020) and E18.5 (*P* = 0.0210), respectively. C57BL/6 mouse placentas with E16.5 and E20.5 days of pregnancy (*P* = 0.0094 and *P* = 0.018,resp.) showed augmented* H2-Q10* levels. Overall,* Aire* transcripts were higher in BALB/c mice compared with C57BL/6 mice, being significantly increased in 10-day spleen (*P* = 0.0242), in E18.5, E20.5, and 15-day gut (*P* = 0.0002, *P* = 0.0067, and *P* < 0.01, resp.), and in E20.5 day placenta (*P* = 0.0009) (data not shown).

### 3.5. Correlation of Gene Expression Profiles during Thymus Ontogeny

Considering the thymus tissue samples obtained at fetal ages, we found a positive correlation between* Aire* and* H2-Q7* in both strains analyzed (*R* = 0.0378/*P* = 0.4025 for C57BL/6, and *R* = 0.03618174/*P* = 0.9638 for BALB/c). Negative correlations between* Aire* and* H2-T23* were found for C57BL/6 (*R* = −0.0408/*P* = 0.9306) and for BALB/c mice (*R* = −0.1852341/*P* = 0.8148). In C57BL/6 mice, the expression of the* H2-Q10* gene showed a positive correlation with* Aire* (*R* = 0.1311/*P* = 0.7793); however, a negative correlation was observed in BALB/c mice (*R* = −0.5067555/0.4932). After birth, negative correlations between* Aire* and* H2-Q7* were observed for both strains (*R* = −0.424/*P* = 0.4016 for C57BL/6, and *R* = −0.1140859/*P* = 0.8076 for BALB/c). A positive correlation between* Aire* and* H2-Q10* and* Aire* and* H2-T23* was observed for C57BL/6 and BALB/c mice (*R* = 0.3409015/*P* = 0.5085 and *R* = 0.8148293/*P* = 0.04826, resp.). However, in BALB/c mice this correlation was negative (*R* = −0.4232537/*P* = 0.3441 for* H2-Q10*, and *R* = −0.1459941/*P* = 0.7548 for* H2-T23*).

## 4. Discussion

### 4.1. MHC Class Ib Genes and the Thymic Selection

Although the* Aire* gene has a well-recognized role in central tolerance, the role of nonclassical MHC molecules, which also have tolerogenic properties, is not fully understood. To evaluate the relationship between* Aire* and* nonclassical MHC class I* genes, we studied the simultaneous expression of these genes during the ontogeny of lymphoid and nonlymphoid organs, from embryonic ages to adulthood.

In the present study, the* H2-Q7* gene (Qa-2 molecule) was abundantly expressed in the thymus compared with other genes and in other tissues at any age analyzed. Several lines of evidence indicate that Qa-2 may be involved in migration, maturation, and effector mechanisms of cells that emigrate from the thymus. Cells exhibiting a high expression of Qa-2 do migrate to the periphery and perform their effector mechanisms [26, 27], corroborating previous results showing that Qa-2 is a marker of medullary thymocytes in the final stages of development [25–27]. In addition, Qa-2 may be involved in the generation of T CD8^+^ lymphocytes specific for antigens that are presented at the periphery in the context of these molecules [12, 26] and in the selection and regulation of CD8**αα**/TCR**αβ** intraepithelial T cells [34].

In humans, the expression of HLA-G in the fetal thymus may be related to the inhibition of thymus NK cells, potentially capable of destroying thymocytes expressing classical HLA class I molecules at low density [35] or even inducing apoptosis of CD8^+^ T cells via Fas expression [8, 28]. Also, the expression of HLA-G by mTECs may induce immune tolerance driven by antigen-specific T cells through the expansion of natural regulatory CD4^+^ Foxp3^+^ T cells [8, 36]. Taken together, these findings indicate that the high expression of* H2-Q7* during perinatal and adult ages may be related to the formation of subtypes of functionally mature thymocytes residing in thymic medulla. The increased expression of* H2-Q7* in adulthood may further indicate that, instead of thymic involution, the presence of mature and functional thymocytes may maintain the functionality of the thymus in thymocyte generation.

It is interesting to observe that the expression of the* H2-Q7* and* Aire* genes exhibited closely similar profiles and showed positive significant correlations in C57BL/6 and in BALB/c mice only during fetal ages. Negative selection is crucial to maintain the homeostasis of the immune system, a process in which the* Aire* gene plays a central role, since it is directly implicated in the control of the expression of thousands of TRAs [21, 22]. Based on the positive correlations between the transcript profiles of* Aire*,* H2-Q7*, and* H2-Q10* in the fetal thymus, we may hypothesize that nonclassical MHC class I genes are also under the transcriptional control of* Aire*. Additionally, it has been proposed that* Aire* may activate genes that are usually silenced or expressed at low levels due to methylation marks (H3K4me0) in their promoter regions. The low expression and the presence of methylation marks may be a feature of* Aire*-dependent activation of genes which are not normally expressed, as in mTECs [23, 24]. In humans, the expression of the* HLA-G* gene is regulated at epigenetic levels due to the presence of methylation of CpG motifs in the promoter region [37, 38] and could also be under the transcriptional regulation of* Aire*.

In contrast to* H2-Q7*, we found a negative correlation between* Aire* and* H2-T23*(Qa-1) expression during fetal thymic development in both mouse strains. Indeed,* H2-T23* transcription levels were reduced during the fetal period of thymus development, increasing during postnatal ages. These results corroborate previous studies reporting that Qa-1 is primarily expressed by antigen-presenting cells and activated lymphocytes during the effector phase of the immune response [4, 11, 12]. The expression of Qa-1 (*H2-T23*) in the fetal thymus, as observed in the present study, may be related to the generation of regulatory T cells, since Qa-1 is involved in the regulation of autoimmunity by suppressive CD8^+^ T regulatory cells. The positive selection of potentially Qa-1-dependent CD8^+^ Tregs may allow the expansion of these regulatory cells in the peripheral lymphoid organs after encountering the cognate antigen [4, 11, 12].

There are no previous studies evaluating the expression of* H2-Q10* in the thymus, and there are few studies reporting the immunomodulatory role of the Qa-10 molecule. In this context, it has been reported that Qa-10 is liver-specific and may be related to better acceptance of hepatic allografts [14, 15]. At least at a transcriptional level, we demonstrated the expression of* H2-Q10* outside the liver. Although a reduced expression of* H2-Q10* during thymus ontogeny was observed in this study, the expression levels were higher during the fetal period. Additionally, we found a significant positive correlation between* Aire* and* H2-Q10* in C57BL/6 mice both for embrionary and for postnatal ages. In contrast, we observed a significant negative correlation between* Aire* and* H2-Q10* in BALB/c mice for embrionary and postnatal ages. As proposed for* H2-Q7*,* Aire* may also be involved in the transcriptional regulation of* H2-Q10*(Qa-10), which in turn may be involved in tolerance in the thymus. Since the expression patterns of* Aire* and* H2-Q10* are distinct in different strains, one may hypothesize that* H2-Q10* expression may be associated with differential immune response patterns, yielding differential susceptibility to foreign or modified self antigens.

### 4.2. MHC Class Ib Genes during Ontogeny of Lymphoid and Nonlymphoid Tissues

Overall, in peripheral organs such as the spleen, liver, and gut, the transcription profiles of* H2-Q7*,* H2-Q10*, and* H2-T23* were characterized by a gradual increase of transcript levels with aging. In the spleen, the increased expression of* H2-Q7* in C57BL/6 mice supports previous studies reporting that the expression of Qa-2 by spleen cells is sufficiently high to prime CD8^+^ T cells [39]. Additionally,* H2*-*T23* transcripts found in this organ may be related to a higher state of activation of T cells, since the expression of Qa-1 preferentially occurs in activated immunocompetent cells [12].

In the liver, both during the fetal period and in adulthood, we observed an increased level of nonclassical MHC transcripts, particularly those encoding the Qa-10 molecule. The increased* H2-Q10* liver transcription rates could serve to modulate immune responses maintaining the “immunosuppressed” state of the liver. This idea is corroborated by studies reporting that hepatocytes expressing Qa-10 are apparently free of autoimmune processes and show little evidence of cell damage [14]. The early expression of MHC class Ib genes may be involved in the maintenance of this “immunosuppressed” state from embryonic stage to adulthood. This maintained state of tolerance in liver cell subpopulations possibly contributes to the high rates of acceptance of liver transplants; however, it may also contribute to liver vulnerability to chronic pathogens, such as hepatitis viruses and* Plasmodium *spp. [40]. We agree with the idea raised by Stroynowski and Tabaczewski in which since Qa-10 appears in the circulation as a soluble molecule, the high expression of Qa-10 would be an additional mechanism by which the liver could impose systemic immunological tolerance, influencing the immune responses at other body sites, particularly in autoimmune manifestations or in allografts [3].

The transcription of the* H2-Q7*,* H2-Q10*, and* H2-T23* genes was detected from E13.5 day during the embryonic development of liver. It is well established that during embryogenesis the fetal liver acts as an important hematopoietic organ, producing diverse cell types as the progenitors of T, B, NK, and dendritic cells and monocytes [41, 42]. Indeed, under normal conditions, these cells and hematopoietic stem cells are able to express Qa-2 and Qa-1 [3, 4]. It has also been demonstrated that fetal liver mesenchymal stem cells express HLA-G molecules [43], and some subtypes of CD8^+^ T cells have been identified as natural CD8^+^ T cells, exhibiting the CD8^+^ HLA-G2^+^ and CD8^+^ CD122^+^ phenotypes [40]. Apparently, the expression of HLA-G in liver cells may contribute to immunosuppression events observed in the liver, favoring the chronification of infections [44, 45].

*H2-Q7* expression in the gut is of potential interest, since several lines of evidence show that Qa-2 is involved in the selection and maintenance of mucosal CD8**αα**/TCR**αβ** intraepithelial lymphocytes and therefore in the regulation of immune responses [39]. It is known that regulatory T cells are abundantly found in the lamina propria of the gut, which can be generated at these sites or may migrate through homing receptors to the gut [46]. Thus, the increased levels of* H2-T23* transcripts observed in the gut may be related to the involvement of Qa-1 in the suppression of NK cell responses and the maintenance and generation of CD8^+^ regulatory T cells, contributing to oral tolerance in the gut [12, 13].

The importance of Qa-1 and Qa-2 in the placenta was demonstrated by studies showing that**γδ** TCR lymphocytes present in the decidua are oligoclonal and restricted to antigens presented by class Ib molecules. Interestingly, these populations of**γδ** T cells are selected in the thymus during the fetal period [47]. The expression profiles of* H2-Q7* and* H2-T23* observed in our study are consistent with human studies showing that the HLA-G molecule has well-marked temporal regulation during pregnancy, with high expression in the first months and decreased expression in the third trimester of pregnancy [47]. Our observation that* H2-T23* transcripts are more expressed* H*2*-Q7* transcripts is surprising, considering that the placenta and embryonic tissues are described as the relevant sites of expression of Qa-2 and a major component of the* H2-Q6/Q7/Q8/Q9* genes (*Ped *gene) involved in preimplantation and embryonic development [6, 9].

Overall, we did not observe expression of MHC class Ib genes in the brain, except for a faint expression of* H2-Q7* and* Aire* transcripts in both mouse strains (data not shown). Under physiological conditions, brain Qa-2 expression has been associated with the development and plasticity of the organ [48]. On the other hand, in humans, induced brain expression of HLA-G has been reported during the course of inflammatory diseases such as multiple sclerosis and has been associated with inhibition of responses mediated by cytotoxic T cells, NK cells, and inhibition of T cell proliferation [46].

The expression of* Aire* transcripts outside the thymus, found here to be reduced in the brain and to occur in considerable levels in peripheral organs such as the spleen and gut, is quite interesting, since in recent years several studies have attempted to identify the expression of* Aire* and the occurrence of PGE in tissues other than the thymus [17, 18, 32]. The relevance and functionality of the expression of* Aire* in peripheral lymphoid organs are still very controversial. Recently, it has been reported that the stromal cells of lymph nodes, spleen, and Peyer's patches express reduced levels of Aire. The expression of Aire by these organs occurs in certain eTACS (*extra thymic Aire-expressing cells*). In a similar way to mTECs, eTACS can perform promiscuous gene expression and are able to mediate deletion of autoreactive T cells [17, 18].

### 4.3. Differential Transcript Profiles between Mice Strains

The comparisons of* Aire* gene expression profiles between strains corroborated our previous studies, showing a peak of* Aire* expression in C57BL/6 thymus at day E16.5 and in BALB/c thymus at day E18.5.* Aire* expression anticipated the PGE phenomenon in both strains and occurred after the beginning of TCR V(D)J recombination. This process starts on the E14.5 day and E16.5 day in C57BL/6 and BALB/c mice, respectively [49, 50]. Therefore, the timing of T cell maturation during thymus development apparently differs between these strains, suggesting an important role of the genetic background in the modulation of these important thymus events [51, 52]. In addition, differences in imunomodulatory MHC class Ib gene expression profiles between strains, as observed in this study, may provide further evidence of the patterns of susceptibility and resistance to infections, autoimmune diseases, and cancers of these strains. In most age groups analyzed in this study, the expression of* H2-Q7* in the thymus and other peripheral organs was significantly higher in C57BL/6 mice than in BALB/c mice. This may reflect the fact that BALB/c mice usually express lower levels of Qa-2 as they present a deletion of both* H2-Q6* and* H2*-*Q9 loci*, and therefore the BALB/c strain is characterized as medium producers of Qa-2 (Qa-2^med^) [3].

Considering that* Q7* and* Q9* genes synergistically contribute to the expression of Qa-2, the genetic differences between BALB/c and C57BL/6 mice may contribute to differential expression of Qa-2, which is approximately 4 to 5 times higher in C57BL/6 mice [3]. In contrast,* H2-T23* and* AIRE* transcript levels were significantly higher in BALB/c mice compared to C57BL/6 mice. Compared to C57BL/6 mice, BALB/c mice are more vulnerable to infections triggered by* Staphylococcus aureus*,* Mycoplasma pulmonis*, and* Leishmania major* [51–53]. Considering that Qa-1 is clearly involved in the generation of regulatory T cells [4, 12, 13] and that* Aire* may shape the repertoire of regulatory T cells [18], increased expression of these transcripts may account for increased central regulatory function and increased susceptibility to infections [54].

## 5. Conclusions

The present study raised the idea of a potential transcriptional link between* Aire* and nonclassical MHC class I genes acting at a central level during thymic education and potentially influencing and modulating the immune responses at the periphery. Although using a generalist approach, this study aimed to characterize the transcription patterns of relevant immunomodulatory genes and may be useful for further studies regarding the involvement of nonclassical MHC class I molecules in immune tolerance events. The evaluation of the protein product encoded by these genes is crucial to understand the relationship and possible transcriptional regulation of nonclassical MHC class I molecules by Aire. Further analysis will be performed in this area in order to verify the potential influence of Aire on the promoter regions of* H2-Q7*,* H2-Q10*, and* H2-T23*. Studies involving appropriate animal models for MHC class Ib molecules can contribute to the current knowledge about HLA-G and HLA-E in humans and are a prerequisite for the development of therapeutic strategies such as the production of nonclassical MHC recombinants molecules associated with immunosuppressive therapy.

## Figures and Tables

**Table 1:** Relative Quantification of gene expression profiles of *H2-Q7*, *H2-Q10*, *H2-T23* and *Aire* throughout the development of C57BL/6 mice. The lines are representative of the mean values of 2^−ΔΔCt^. Tissue samples were obtained in triplicate from different animals for each age analyzed. Each experiment was independently performed at least three times. Data were analyzed statistically by One-way ANOVA followed by the Bonferroni multiple comparison test. The comparison was performed between the transcript levels of one target gene in the same tissue but at different ages. Values close to the level of significance are marked with (∗). Values near the significance level (*P* < 0.05) are marked with (†), (*P* < 0.01) with (•) and (*P* < 0.001) with (∗). was utilized.

	Thymus				Liver				Intestine			
	*H2-Q7 (Qa2) *	*H2-T23 (Qa1) *	*H2Q10 (Qa10) *	*Aire *	*H2-Q7 (Qa2) *	*H2-T23 (Qa1) *	*H2Q10 (Qa10) *	*Aire *	*H2-Q7 (Qa2) *	*H2-T23 (Qa1) *	*H2Q10 (Qa10) *	*Aire *
E14.5	0.0102	0.3810	0.0344	0.1617	0.1355	0.0735	0.0187	0.0000	0.0515	0.0080	0.0365	0.1550
E15.5	0.0966	0.1100	**0.1967** ^†^	0.2500	0.1465	0.0725	0.0286	0.0000	0.0340	0.0045	0.0365	0.2893
E16.5	0.0920	0.2220	0.6565*	0.9500*	0.088	0.1447	0.0765	0.0000	0.0333	0.0203	0.1503	0.2180
E17.5	0.0785	0.3020	0.0200	0.9850*	0.09	0.1160	0.0330	0.0000	0.0426	0.0156	0.1273	0.7205
E18.5	0.0066	0.2060	0.0172	0.1075	0.1008	0.1535	0.1156	0.0000	0.0246	0.0296	0.2140	0.3527
E19.5	0.1070	0.2300	**0.3683** ^†∗^	0.1367	0.0625	0.1330	0.0956	0.0000	0.0185	0.0355	**0.7255** ^†•∗^	0.3760
E20.5	0.0560	0.3540	0.5505*	0.0200	0.112	0.1308	**0.1775** ^†^	0.0000	0.0280	0.0660	**0.949** ^†∗^	0.3000
1	**0.1645** ^†^	**0.4323** ^†•^	0.9955*	0.0733	0.0625	0.2020	0.0370	0.0000	0.0267	0.1137	0.2960	0.2175
5	0.0243	**0.6658** ^†•∗^	0.5250*	0.1700	**0.2225** ^†^	**0.6850** ^•∗^	0.0917	0.0000	0.0695	0.1997	0.2400	**0.7715** ^†^
10	0.0166	0.3185	0.0172	0.0200	**0.332** ^•∗^	**0.6167** ^†•^	0.1347	0.0000	0.1900	0.4580	0.0080	0.3810
15	0.0320	**0.6668** ^•∗^	0.0423	0.1850	0.185	**0.6743** ^†•∗^	0.1513	0.0000	0.1905	**0.5445** ^†^	0.0230	0.2750
45	0.2190^†^	**0.4633** ^•^	0.0563	0.0866	0.9995*	0.6380^†•^	0.7717*	0.0000	**0.6305** ^•∗^	0.3805	0.0530	0.3080
60	0.9158*	0.2993	0.0425	0.0360	0.91*	0.8385*	0.5723*	0.0000	0.9175*	**0.699** ^†•∗^	0.0360	0.1310

	Spleen				Placenta				Brain			
	*H2-Q7 (Qa2) *	*H2-T23 (Qa1) *	*H2Q10 (Qa10) *	*Aire *	*H2-Q7 (Qa2) *	*H2-T23 (Qa1) *	*H2Q10 (Qa10) *	*Aire *	*H2-Q7 (Qa2) *	*H2-T23 (Qa1) *	*H2Q10 (Qa10) *	*Aire *

E14.5	—	—	—	—	0.0326	0.2435	**0.7957** ^†^	0.0210	0.0580	0.0350	0.0170	0.6820
E15.5	—	—	—	—	0.0450	0.6507	0.2425	0.0596	0.1823	0.0230	0.0330	0.7630
E16.5	—	—	—	—	0.0413	0.3227	0.2490	0.0693	0.1673	0.0060	0.0370	0.3703
E17.5	—	—	—	—	—	—	—	—	0.1453	0.0185	0.0170	0.2370
E18.5	—	—	—	—	—	—	—	—	0.1570	0.0140	0.0150	0.2220
E19.5	—	—	—	—	—	—	—	—	0.1375	0.0120	0.0535	0.3280
E20.5	—	—	—	—	0.0233	0.2803	0.0430	0.8767*	0.3205	0.0140	0.6965	0.6355
1	0.0165	0.1047	0.2103*	0.0295	—	—	—	—	0.3030	0.0000	0.0855	0.4105
5	0.0085	0.0965	0.0190	0.0836	—	—	—	—	0.0000	0.0880	0.0140	0.1940
10	0.0343	0.2217	0.0126	0.0126	—	—	—	—	0.6560	0.0510	0.0270	0.1980
15					—	—	—	—	0.0440	0.0050	0.0330	0.2750
45	0.1667	0.5817	0.0533	0.0806	—	—	—	—	0.1110	0.0250	0.0530	0.1820
60	0.7665*	0.5973	0.0553	**0.2073** ^•∗^	—	—	—	—	0.3530	0.0400	0.1165	0.1355

^†^(*P* < 0.05); ^•^(*P* < 0.01); *(*P* < 0.0001).
